# Ants engaged in cooperative food transport show anticipatory and nest-oriented clearing of the obstacles surrounding the food: goal-directed behavior emerging from collective cognition

**DOI:** 10.3389/fnbeh.2025.1533372

**Published:** 2025-06-13

**Authors:** Ehud Fonio, Danielle Mersch, Ofer Feinerman

**Affiliations:** Department of Physics of Complex Systems, Weizmann Institute of Science, Rehovot, Israel

**Keywords:** ants, collective obstacle clearing, cooperative transport, anticipatory behavior, emergent cognition, ethology and behavioral ecology, superorganism

## Abstract

One of the hallmarks of higher cognition is the ability to anticipate near-future events and effectively react to them. This requires perceiving events in a dynamic environment and adjusting the actions accordingly to suit the expected outcomes. Social insects exhibit various forms of emergent collective cognition; however, it is not clear whether such preplanning is one of them. We discovered that when longhorn crazy ants cooperatively carry a large food item to the nest, some ants clear the path ahead of the moving load from small debris. The obstacle clearing is nest-oriented, as it creates a clear path connecting the food load with the nest. We show that this anticipatory obstacle-clearing behavior is context specific and that it is functional in reducing the time needed to deliver the large food load to the nest. Importantly, we found that no personal knowledge of the food load is required for the ants to start clearing the obstacles. Individual ant tracking revealed that clearing is instead triggered by social cues in the form of freshly laid pheromone markings. Indeed, we observed that obstacle clearing was performed by ants that had never experienced the big food load and even in cases where no such load was present at all, in response to the pheromone marks alone. These results provide strong evidence that individual ants do not possess an internal representation of the final goal of obstacle clearing. On the other hand, the goal-directedness of obtacle clearing appears to emerge at the ant group level from collective cognition.

## 1 Introduction

Animals exhibit anticipatory behaviors (Talley et al., 2023; Lindauer, 1960; Weir et al., 2002; Hopcraft et al., 2005; Menzel and Manz, 2005; Miyata, 2021; Perry and Chittka, 2019) that allow them to prepare for near-future events and respond before they occur (Emery and Clayton, 2004). This complex behavior often relies on internal representations (Pezzulo, 2008), which allow the animal to incorporate sensory information, memory, predictive modeling, and decision making (Bubic et al., 2010; Miyata, 2021). In this sense, internal representations allow the animal to extend its actions beyond the here-and-now of the sensory-motor cycle. This makes anticipatory behaviors a hallmark of advanced cognition (Miyata, 2021; Craik, 1967).

Groups of ants display behaviors that may have an anticipatory component. For example, ants pave sticky terrains with sand to create safe passages to a food source before it is reached (Wen et al., 2021). Another example occurs when ants clear trails to food sources of debris and vegetation allowing a speedier traversal (Middleton et al., 2019; Plowes et al., 2013; Rockwood and Hubbell, 1987; Howard, 2001). Trail clearing is a slow process exhibited by many ant species and reported to occur on the order of several days or more (Middleton et al., 2019). As such, it is associated with long-lasting trails to stable food sources or trunk trails toward specific areas (Howard, 2001; Bochynek et al., 2017; Rockwood and Hubbell, 1987). There is evidence that trail-clearing ants are part of a dedicated subgroup and that each may engage in multiple clearings (Bochynek et al., 2019; Howard, 2001). However, there is no evidence for what social information is required to trigger clearing behavior (Bochynek et al., 2019). Finally, although trail clearing occurs concurrently with foraging, its energetic benefits also affect future trail use (Howard, 2001). Taken together, these behaviors provide signs for anticipation by the ant colony. Importantly, in current scientific literature there is almost no evidence by which to discern whether this anticipation originates from single ants working to resolve their personally experienced difficulties (which may even include creating an internal representation of the future path of the load) or as an emergent group-level phenomenon.

Social insects exhibit decentralized collective cognition (Sumpter, 2006; Couzin, 2009; Feinerman and Korman, 2017). Examples include collective decision-making, collective navigation, and flexible task allocation. Interestingly, some of these rely on external analogs of internal representations. Ants, for example, can alter their environment (stigmergy) to represent memory (Theraulaz and Bonabeau, 1999; Heylighen, 2016). Indeed, using pheromones to mark the route to a food source relieves individual ants of remembering the route and can be viewed as an external representation that is etched onto the environment itself. Such pheromone markings can be used to encode short- and long-term memories (Dussutour et al., 2009) and aid in decision making about the most efficient route to food (Czaczkes et al., 2015; Correia et al., 2017). It is not known whether such representations can be employed to support anticipatory behaviors on the colony scale.

**Figure 1:**
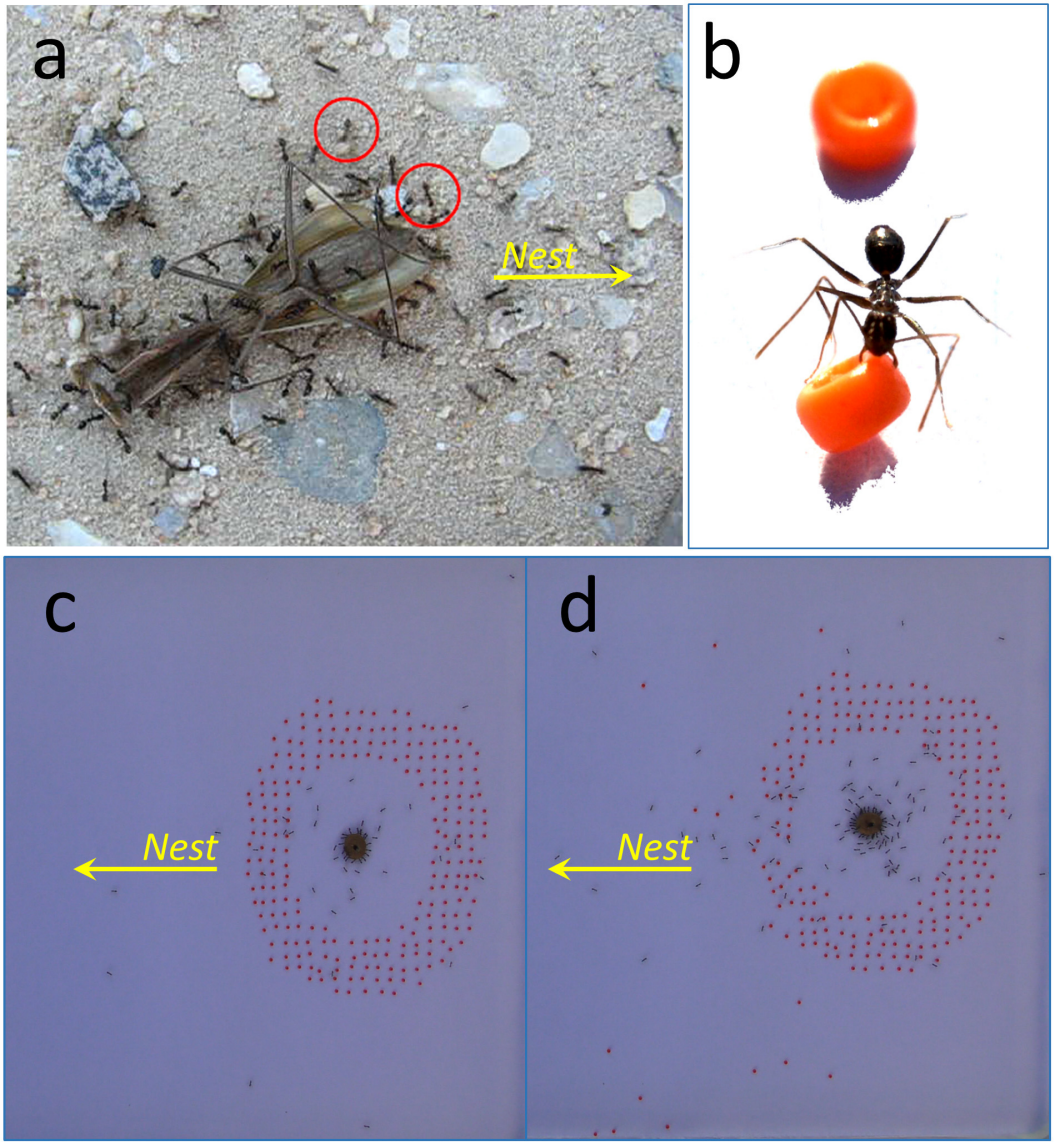
Obstacle clearing behavior: **(a)** A group of ants during natural cooperative transport of a mantis carcass. Two ants, marked by red circles, are clearing small gravel. See also Supplementary Video S1, recorded upon discovery of this phenomenon, from which this picture was taken. **(b)** An ant is clearing a bead in the experimental setup. For clarity, the image was manipulated to remove shadows. The original picture is given as Supplementary Figure S9. **(c)** A top view of an experimental set-up in which a food pellet was placed on a pin and surrounded by a ring of red beads in the testing area. The picture was taken before any bead was cleared. **(d)** A snap-shot from the same experiment as in **(c)** after about 15 min during which several beads were cleared.

When ants find a food load that is too big to be carried individually, they engage in cooperative transport (Czaczkes and Ratnieks, 2013; McCreery and Breed, 2014; Gelblum et al., 2015). In natural environments (Figure 1a) the surface is often riddled with fine gravel whose pebble size is comparable to that of an individual ant. In the present work, we studied cooperative transport by the longhorn crazy ants (*Paratrechina longicornis*) (Trager, 1985; Wetterer et al., 1999; Wetterer, 2008; Feinerman et al., 2018) in such environments. We report here for the first time the anticipatory obstacle clearing behavior of free-living longhorn crazy ants, documenting it with videos. Basically, during cooperative transport, while some ants hauled the load, some other ants cleared small gravel from the vicinity of the moving load (see our first observation of this phenomenon in Supplementary Video S1). While some pebbles were removed directly under the load, others were removed from locations that were far away from it. These distant pebbles, often located on the path connecting the load and the entrance to the ants' nest, were hence removed well before they had any direct effect on the moving load. The ants that engage in this clearing behavior appear as if to anticipate the future trajectory of the load and preemptively clear it of possible obstacles that can slow down the retrieval process. This behavior is anticipatory as the ants clear the debris before it physically interferes with the passage of the food load. Notably, the findings reported in this work represent the first ever observation of debris clearing in the context of cooperative transport of large food loads by ants. In what follows, we report this newly discovered anticipatory clearing behavior and we investigate the possible mechanisms inducing it. In particular, we explore whether anticipation may originate at the individual level of the single ants or rather emerge at the level of the ant group as a result of collective cognition.

## 2 Methods

### 2.1 Experimental set-up and data acquisition

**Figure 2:**
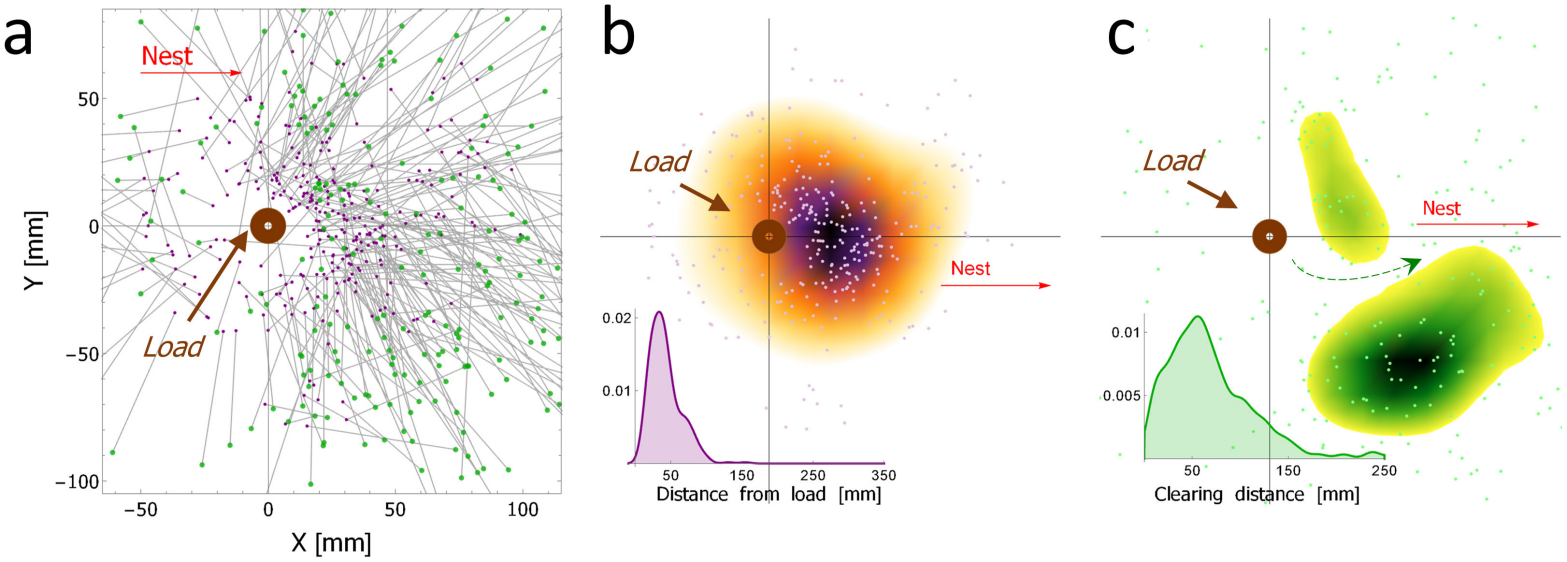
The spatial properties of clearing behavior: **(a)** The overall distribution of cleared beads (*N* = 292 from 25 type 1 experiments) with respect to the food load (the round brown ring positioned at {0,0}), including both their original position (purple dots), and the final position (green dots) after the bead was dropped by the ant, and connected by a gray line to the corresponding origin. To reduce the visual load on this graph, we separated the original and final positions of the beads and displayed the density of each set separately while preserving the original data (pale dots): **(b)** The spatial density of the original bead positions [purple dots in **(a)**]. The inset shows the density distribution of the distance-from-load for the original bead positions. **(c)** The spatial density of the final position of the cleared beads after they were dropped [green dots in **(a)**]. The inset shows the density distribution of the clearing distances. The green arrow denotes the relatively free passage in the nest direction that was created by the clearing activity.

To adequately measure the collective clearing behavior of the longhorn crazy ants, we devised a standardized experimental setup in which natural, irregular, small gravel (Figure 1a) was replaced by uniform colored beads [MIYUKI 15/0 1.5 mm round seed beads 406 (orange), Figure 1b]. The beads are similar to the common gravel size that the ants were observed to clear under natural conditions (Supplementary Video S1), although occasionally the ants were also observed to clear larger obstacles (see an extreme example in Supplementary Figure S1, Supplementary Video S2). Various colors were tested, but no preference was observed by the ants; therefore, we used bright orange beads, which are more easily detected via image processing. In our preliminary observations, we observed that the load often pressed on the gravel just before the latter was cleared. In such cases, the ants may have cleared the gravel merely because it was smeared with food. To focus on gravel that is removed for clearing purposes, we prevented direct contact between the beads and the load by pinning the large load to the floor of the experimental setup (this holds for most of the configurations used). The experimental setup included an enclosed arena (30 × 25 cm) that was placed on the ground approximately 1 m from the main entrance of the natural nest, so that the ants were allowed to freely enter the arena through an opening in the wall facing the nest. Marks on the edges of the arena (Supplementary Figure S2) were used for calibration, allowing us to measure the positions of the beads and their clearance distances (as in Figure 2, for example). All experiments were performed outdoors during the summer, on a supercolony of *Paratrechina longicornis* distributed at the Weizmann Institute of Science, Israel. The experiments were recorded using a Panasonic camcorder (model HC-VX870) at a 4K resolution and 25 frames per second. A whole cat food pellet (Royal canin FIT) or a silicon ring that was immersed in the same cat food brand were placed inside the arena and used as bait. Previous studies on these ants and this type of food showed that the ants do not care much about the shape of the food and both loads used here induced similar cooperative transport behavior (Gelblum et al., 2015; Fonio et al., 2016) as well as collective obstacle clearing behavior. The delivered food bait was either fixed by a pin, preventing the ants from carrying it away and over the beads, or without a pin, enabling the execution of cooperative transport (see more details about specific experiments below). The pinned loads could still rotate around their axis to maintain a high level of interest from the ants in the load. The experiments lasted at least 30 minutes, in which clearing events usually began during the first 10 min. To study the relevant aspects of collective clearing behavior, various experimental conditions were used. Further specifications of the experiments are given below.

### 2.2 The experiments

#### 2.2.1 Experiments type 1: the spatial properties of the clearing behavior

A food load was placed on a pin, allowing rotation only, in an experimental set-up with beads scattered on the floor as close as 2 cm from the load (Supplementary Figure S3). We analyzed 292 bead-clearing events, from 25 such experiments, and we visualized the results in graph through gray lines connecting the original position (a purple dot) and the final position (a green dot) of the bead after it was dropped by the ant (Figure 2a). Subsequently, we created two further graphs representing the original and final positions, respectively (Figures 2b, c). To further clarify these two plots, we used a cut-off value, showing only the highest densities (ρ > 0.00003). We used the exact same threshold, so the densities in the two plots are comparable. The color gradient reflects the density level from bright (low density) to dark (high density). Based on the results about the typical clearing distance (inset of Figure 2b), in some of the following experiments a ring of beads was used around the load rather than covering the whole experimental area (as can be seen e.g., in Figures 1c, d). The ring ranged between 4 cm (innermost distance) and 8 cm (outermost distance) around the load.

#### 2.2.2 Experiments type 2: clearing function

**Figure 3:**
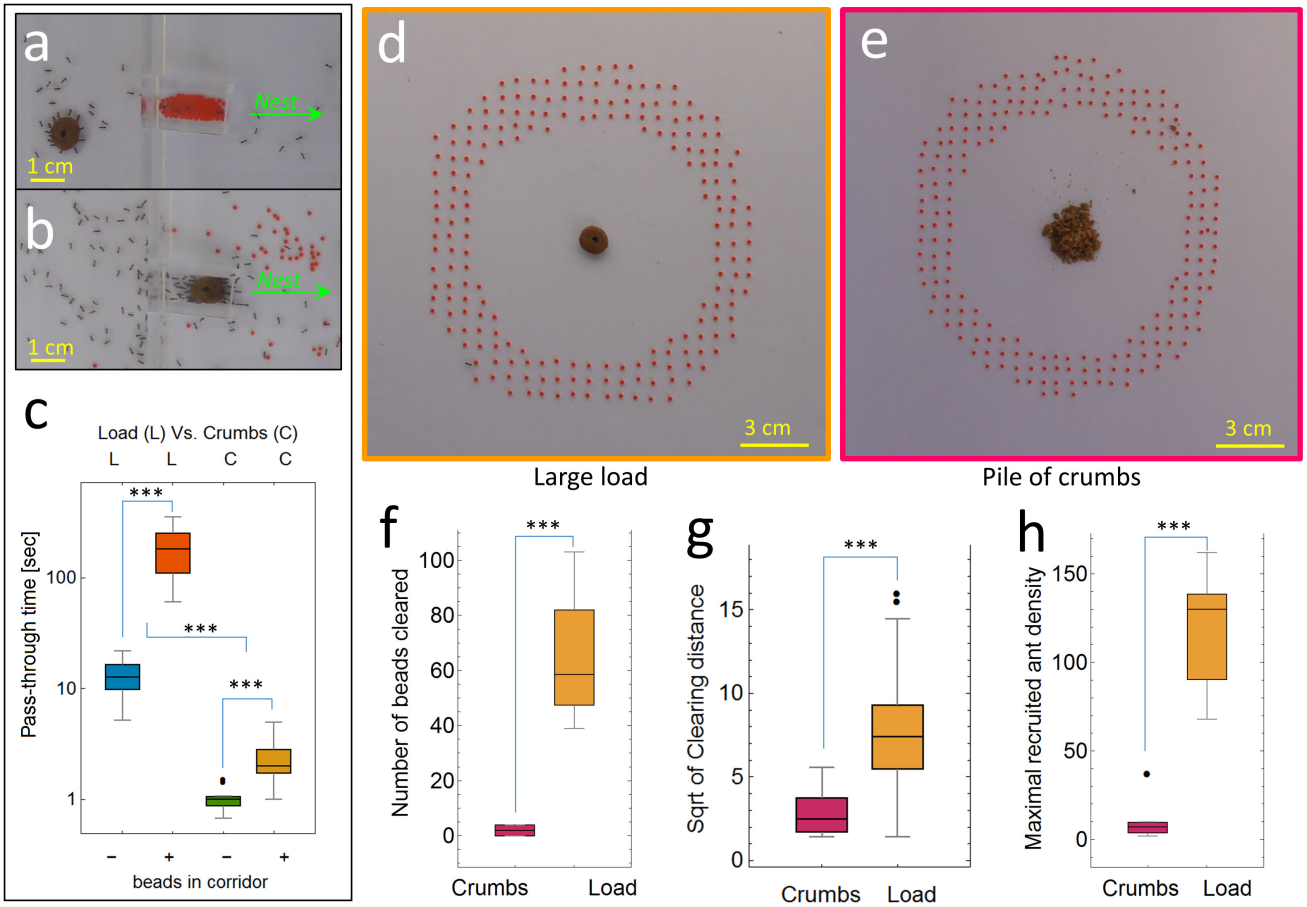
Left—Clearing function (type 2 experiments): **(a)** a load (brown object) blocked by a corridor full of beads, **(b)** load transport after beads were cleared from the passage. Green arrows denote nest direction. **(c)** Load pass-through time (in seconds, log scale) through either an empty corridor (crumbs: *N* = 12, median = 1, SD = 0.2 and load: *N* = 14, median = 12.8, SD = 4.7) or a passage full of beads that the ants had to clear first (crumbs: *N* = 14, median: 2.0, SD = 1.0 and load: *N* = 18, median = 182.3, SD = 80.8). Right—Context comparison of clearing behavior between 2 conditions (type 3 experiments): **(d)** a single large doughnut-shaped food item placed on a pin (4 experiments in which a total of *N* = 259 beads were cleared) and **(e)** a pile of crumbs from the same food type (6 experiments) in which only *N* = 12 bead in total were cleared. **(f)** Number of obstacles cleared (crumbs: median = 2, SD = 1.9 and load: median = 58.5, SD = 27.2). **(g)** Clearing distance of obstacles (crumbs: median = 6.5, SD = 8.7 and load: median = 55, SD = 47.9). **(h)** Maximal recruited ant density around the food (crumbs: median = 7, SD = 12 and load: median = 130, SD = 35.9). Asterisks highlight significant differences (*** *p* < 0.001).

We analyzed a total of 32 dedicated experiments, where we used a narrow (1 × 1 × 3 cm) corridor as a bottleneck, using 4 conditions: (i) cooperative transport of a large load through an empty corridor (*N* = 14). (ii) a large load and a corridor filled with 70–75 beads (*N* = 18). In this extreme case, the ants had to remove most of the beads before they could transport the load through the narrow passage (Figures 3a, b). To compare the effect of obstacle presence on individual vs. cooperative transport, we repeated the above-mentioned experiments but replaced the single large load with crushed food pellets of the same brand. In this way, the ants individually carried the crumbs, so no cooperative transport was needed. The crumbs were of the size of ants or smaller (as in Figure 3e). The following conditions were also tested: (iii) individual transport of food crumbs without beads in the corridor (*N* = 12), and (iv) individual transport of crumbs through a corridor filled with beads (*N* = 14). We compared the duration it took the ants to transport the food across the corridor (defined as “pass-through time”) starting from the first time the food was at least partially in the corridor and until it was fully out on the other side (Figure 3c). In addition, to examine how load transport is being stalled due to the direct interactions with scattered obstacles, we analyzed another set of tests in which we measured the pass-through time of a large load through a 5 × 7 cm passage, either empty (*N* = 8) or scattered with pinned beads (*N* = 7, Supplementary Figure S5). Similarly to the former experiment, here we also compared the pass-through time of ants individually carrying small crumbs (Supplementary Figure S5b).

#### 2.2.3 Experiments type 3: context sensitivity of the clearing behavior

We analyzed a total of 10 dedicated experiments, in which beads were placed as a ring surrounding a single large load (4 experiments in which a total of *N* = 259 beads were cleared) or a pile of food crumbs (6 experiments, in which only *N* = 12 beads were cleared in total). We compared 3 measures: (i) the number of beads intentionally removed by grabbing and pulling the bead with the mandibles (Figures 1b, 3f). (ii) The clearing distance, measured as the distance between the original and final positions of the cleared bead (Figure 3g). (iii) The maximum recruited ant density (Figure 3h), measured by counting the maximum number of ants within a radius of 8 cm around the food, an area that includes the ring of beads (Figures 3d, e), during the first 10 min of the experiment. After confirming the strong relationship of the presence of pheromone marks with the triggering of bead clearing, we examined the recruitment behavior of ants returning from food. To further examine the effect of this difference in recruitment behavior on the rate of ant-to-ant interaction, we reviewed recorded experiments and visually tracked 31 ants (*N* = 14 in the single large load context, and *N* = 17 in the pile of crumbs context), for 10 seconds after entering the experimental setup, and counted the number of interactions with other ants while moving toward the food.

#### 2.2.4 The overall dynamics of the unfolding behavior during clearing experiments

**Figure 4:**
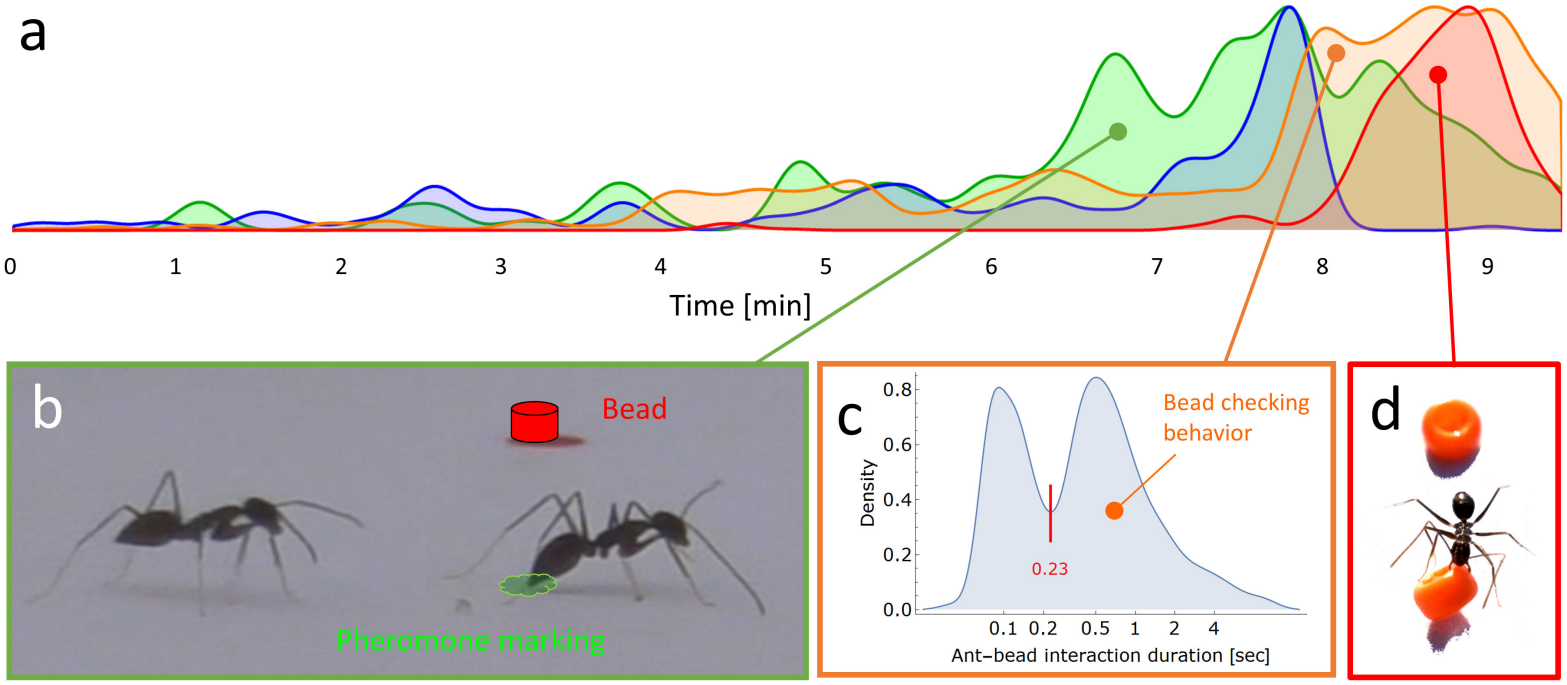
**(a)** A normalized density plot summarizing the intensity along time of the different behaviors that are fully coded in Supplementary Figure S6: pheromone marking (in green), number of ants in the load area (in blue), bead checking (in orange) and bead clearing (in red). **(b)** A recruiter ant displays marking behavior (pheromone is illustrated in green) adjacent to a nearby bead (in red) and to another ant. **(c)** Distribution of ant-bead interaction time (*N* = 932 events) in log scale. Checking bead behavior is defined by the right component in the distribution, representing relatively prolonged checking time of more than the 0.23 seconds threshold denoted by the red line. **(d)** A picture showing an ant removing a bead by grabbing it in its mandibles.

A representative type 1 experiment (Section 2.2.1) was thoroughly coded, including the arrival time of the ants during recruitment and the relevant behaviors they displayed during their visit to the experimental setup, including pheromone markings and ant-bead interactions. This detailed coding, presented in Supplementary Figure S6, was performed only once, just to provide an example of the richness and complexity of this scene. However, this overall dynamics (presented there and in Figure 4a) was typical for the other experiments. This data was also used to analyze: (i) interactions between ants and beads (*N* = 932 events) to distinguish between occasional brief touch with beads which practically did not affect ant motion, and significantly longer interactions that were termed “bead checking” (Figure 4c), and (ii) the typical marking frequency during recruitment as explained in the subsection: “Recruitment and scent mark identification” below).

#### 2.2.5 The microscopic mechanism of clearing triggering

**Figure 5:**
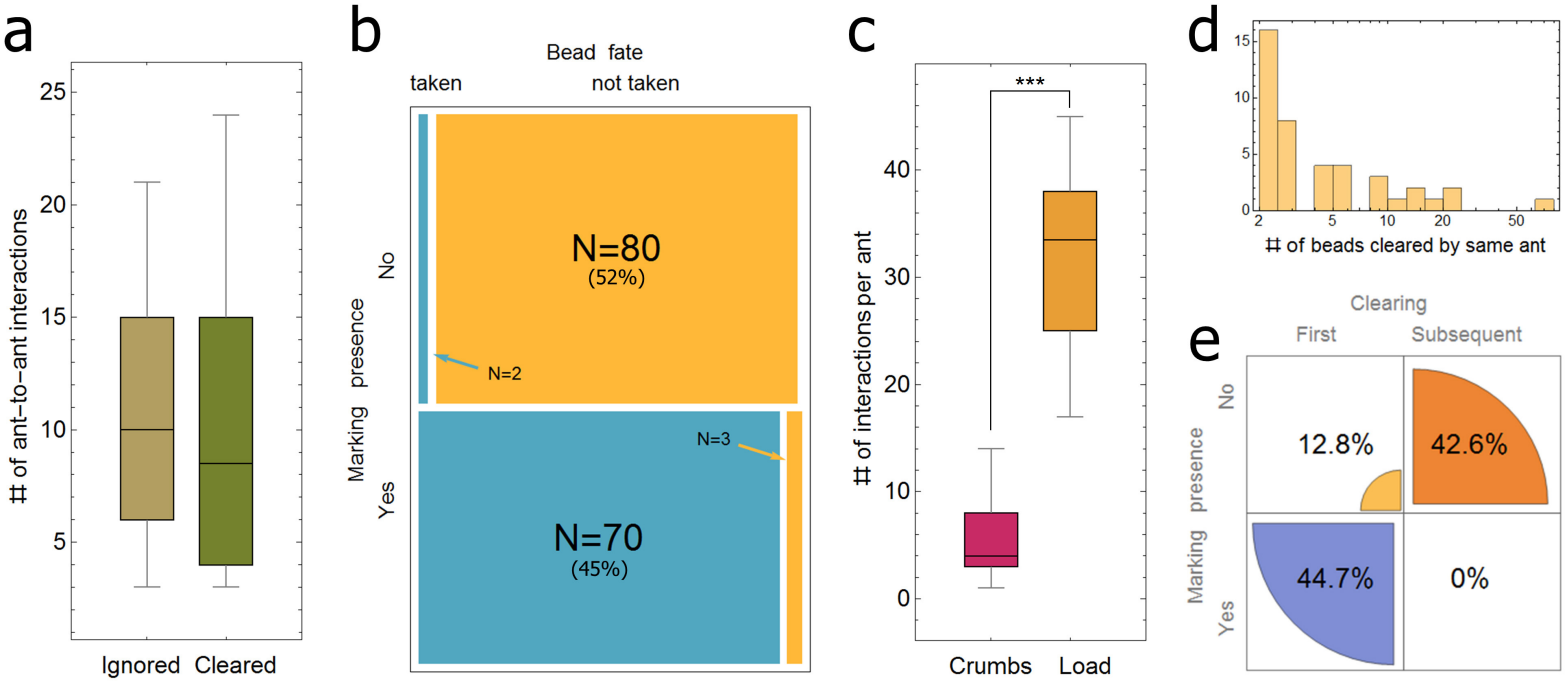
Induction of clearing behavior. **(a)** The number of interactions with other ants during a 10 seconds interval prior to an interaction between this ant and a bead, where the bead was either cleared (*N* = 14, median = 8.5, SD = 6.5) or not (*N* = 13, median=10, SD = 5.8). **(b)** A 2 × 2 matrix of the presence of pheromone marks and the decision to either clear (*N* = 72) or not (*N* = 83). **(c)** The number of interactions between a new ant that entered the experimental set-up and other ants, during a 10 seconds interval, in either the context of pile of crumbs (*N* = 17, median = 4, SD = 4.0) or a single large load (*N* = 14, median = 33.5, SD = 8.0). **(d)** A histogram of the number of beads successively cleared by a single ant. Note that here only data for ants that cleared more than 1 bead are presented (*N* = 42 out of the 167 inspected ants, that cleared altogether 289 out of the total 414 beads cleared). The full distribution is given as Supplementary Figure S7. **(e)** Sequential clearings and marking presence. A 2 × 2 matrix of the order of clearing behavior (1st clearing vs. later clearings by same ant) and its association with the presence of close-range pheromone marks. Asterisks highlight significant differences (*** *p* < 0.001).

For studying what triggers clearing behavior, we conducted two different examinations (based on type 1 experiments as described above): (i) To test the hypothesis that the sensitivity to the density of ants triggers clearing behavior, we reviewed recorded experiments and visually tracked 27 ants that recently entered the experimental setup. We counted the number of interactions these ants experienced with other ants during a time window of 10 seconds before interacting with a bead and making a decision to remove the bead (*N* = 14) or not (*N* = 13). The examination was restricted to direct contact of the head or antenna of the focal ant with other ants (see Figure 5a). (ii) To test the hypothesis that pheromone marks trigger clearing behavior, we observed 155 ant-bead interactions and examined the presence of pheromone markings (as described below) just before the first clearing of a bead by a new ant visiting the experimental setup. The examination was restricted to a radius of 20 mm (about seven times the length of an ant) around the relevant bead position and for 2 seconds prior to the moment when the ant came into first contact with the bead. These criteria are well within the detection range of this ant species (Witte et al., 2007; Fonio et al., 2016) and ensured that our inspections were carried out before any decision (either remove or leave) was apparent to the observer. To prevent bias in the analysis, a blind test approach was adopted in which one of the authors selected a set of events of ant-bead interaction. About half of these eventually resulted in bead-clearing behavior (*N* = 72), whereas the rest did not (*N* = 83). Another author, who was naive about the fate of these beads, then examined all cases in random order. The identification of scent marks in this work was similar to the way it was carried out in previous studies as described in Fonio et al. (2016). In short, recruitment behavior in these ants involves a series of frequent stops ranging between 4 and 7 Hz (unpublished data), where the ant lowers her gaster, briefly touching the floor, and secretes trail pheromone marks. From the detailed coding of one of the experiments (see ethogram in Supplementary Figure S6) we measured the distribution of the time intervals between successive marking events (*N* = 2,351, Supplementary Figure S8). The reciprocal of the commonest value (0.18 seconds) is about 5.5 Hz, confirming the typical marking rate mentioned above. This behavior can be seen directly from the video or indirectly from the recruiter speed profile, where marking events appear as frequent “dips” [see Figure 1 in Fonio et al. (2016)]. In addition, the marking density along the recruitment trail is higher near the load (see Supplementary Video S3 for an example).

#### 2.2.6 Serial clearers

To study the properties of subsequent clearing events by the same ant, another group of 167 ants from type 1 experiments (Section 3.2.1), clearing a total of 414 beads, was tracked and the number of beads subsequently cleared by each was scored (Figure 5d). Ants were visually tracked frame by frame as long as they were confidently identified. Note that this distribution presents only ants that cleared more than one bead (*N* = 42 out of 167 ants, clearing 289 out of 414 beads). The full distribution is shown in Supplementary Figure S7. In addition, these 42 ants were further inspected for the presence/absence of a nearby pheromone marking just before clearing a subsequent bead (Figure 5e).

#### 2.2.7 The spatial properties of repeated clearing behavior

**Figure 6:**
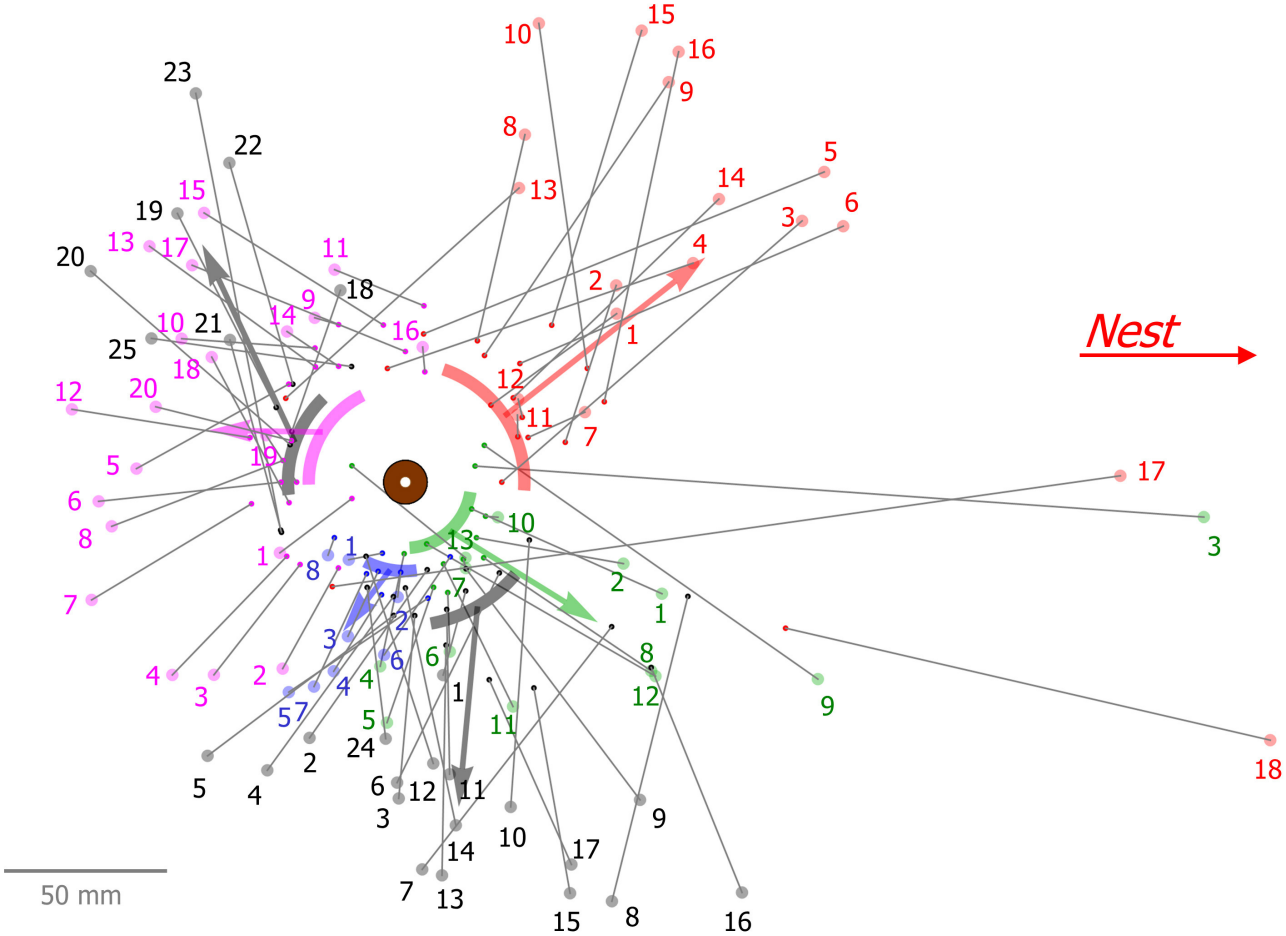
Spatial properties of successive bead clearing by five ants from the same (type 1) experiment. Each color represents beads that were cleared by the same ant, where the small dot is the original position of the bead and the large dot connected by a line is the final position of this bead after it was dropped by the ant. Numerals denote the order of clearings of each ant. Arcs represent the mean and standard deviation, as well as mean distance of the original bead angles, relative to the load, and the arrows show the general direction of bead removal by connecting the mean value of the original and final bead positions.

Here we used a single type 1 experiment, in which 5 ants that repeatedly removed beads cleared a total of 83 beads (Figure 6). The fact that all these events took place on the same scene allowed us to highlight some of the typical properties of serial clearers.

### 2.3 Data and statistical analysis

All data analysis, including visualizations, was performed using Mathematica software (V13.2) by Wolfram Research, Inc. For comparisons between two distributions, we applied the built-in Mathematica function—“TTest” (see Figures 3f–h, 5a, c). In some cases a simple transformation (square root) of the data was needed to achieve the prerequisite normality test (performed automatically in the built-in hypothesis test functions in Mathematica). For clarity, the data presented in the box plots are the original (non-transformed) data; however, the reported p-values in the results section are for the transformed data. For the “pass-through time” results (Figure 3c) we performed a two-way analysis of variance by applying the built-in “ANOVA” function in Mathematica using two factors: food type (a single large load vs. pile of crumbs) and corridor condition (free or filled with beads). To test the association between pheromone marks and clearing behavior (as in Figures 5b, e) we applied Fisher's exact test (Fisher, 1970) which is a recommended test for analyzing contingency tables.

## 3 Results

### 3.1 Collective obstacle clearing

To study the newly discovered obstacle clearing behavior, we tracked the cooperative transport of large loads of food in the field using a number of experimental setups (see the Methods Section) in which we replaced natural gravel with artificial beads (Figure 1b) placed uniformly around the load (Figure 1c). We found that within 15 min after initial recruitment, the ants typically cleared many of these distant beads (Figure 1d).

For bead clearing to be useful, it is necessary not only that beads be picked from the anticipated future trajectory, but also that they be dropped away from this trajectory. To test this, we placed a pinned load in the center of an arena with a large number of beads (see Supplementary Figure S3). We observed a total of 292 clearing events from 25 experiments, as depicted in Figure 2a. The spatial properties of the original positions of the beads selected by the ants for removal reveal a strong preference for clearing beads located about 40 mm away from the load (inset of Figure 2b) and toward the direction of the nest (Figure 2b). Generally speaking, the ants transported these beads for relatively short distances of about 50 mm (inset of Figure 2c). The beads were then removed from the main path creating a lower obstacle density passage between the load and the nest (marked by the green arrow in Figure 2c). Taken together, these results show that the removal of the beads by the ants is mostly limited to the creation of a free passage in the load-nest trajectory, suggesting an anticipation of the immediate future trajectory of the load and goal-directed behavior.

Importantly, our observations show that the ants do not remove beads because they confuse them for food. First, ants identify food by its scent (Provecho and Josens, 2009), but these beads do not carry such scents (and were completely ignored if no food was present in the setup). Second, the distance to which the beads were removed is much smaller than the distance through which the actual food crumbs were carried. In fact, food crumbs are typically delivered all the way to the nest, while beads are dropped after short distances and in various directions (Figure 2). Finally, large food loads induce cooperative transport in which the ants team up to completely surround the load (Gelblum et al., 2015). For the standardization of this study, we used relatively small beads as obstacles. However, in natural conditions, ants were also observed to remove larger objects. In contrast to large food loads, large obstructions never induced cooperative transport and were always transported by a single ant (see Supplementary Figure S1, Supplementary Video S2 for an extreme example). All of the above suggests that bead clearing is a distinct behavior and we next test for its possible functionality.

### 3.2 Clearing function and context sensitivity

To demonstrate the biological functionality of beads clearing, we compared the time it took the load to pass through a corridor that was either empty or full of beads (Experiments type 2 in the methods, Figure 3a). The beads were arranged so that individual ants could still move through the corridor and get to the food. On the other hand, the load could not be transported through the corridor before it was mostly cleared of beads (Figure 3b). The presence of beads caused a 15-fold increase in the time it took the load to pass through the corridor (median of 182.3 seconds with beads vs. 12.8 seconds in their absence, Figure 3c, orange vs. blue box plots, *p*-value < 3*10^−16^). Note that in this experiment, bead clearing ants effectively transferred the configuration of the corridor from “full of beads” to “bead-free” and thus enabled the passage of the load. Supplementary Figure S4, demonstrates this causal relationship by showing that once the corridor is cleared, the load passes quickly, on the order of ten seconds, which is in agreement with the pass-through time in empty corridor (Figure 3c in blue). This means that the measured delays were not caused by the decreased speed of the ants as they traveled over the beads but rather due to the fact that the ants could not transport the load through the corridor before most beads were cleared. To examine the influence of small obstacles on pass-through time in a more direct way, we performed another set of experiments in which we pinned beads in a 5 × 7 cm passage (Supplementary Figure S5a) and as before we compared the cooperative transport of a large load in this condition and in the same passage which is bead-free. This extreme example provides a clear demonstration of the usefulness of bead removal for efficient food retrieval.

Whereas crazy ants transport large food items as a group, they retrieve small food items as individuals. We used the same configuration of corridor and beads but replaced the large food with a pile of crumbs small enough to be manageable by individual ants. We found that the time it took a laden ant to pass through the corridor was significantly lower than the time it took the large load to do the same (ANOVA, “load-type” factor: *p*-value < 8*10^−23^ Figure 3c). This was also true for the presence/absence of beads (ANOVA, “beads-presence” factor: *p*-value < 2*10^−37^ Figure 3c). To examine the influence of small obstacles on pass-through time in a more direct way, we performed another set of tests (see experiments type 3 in the methods section) in which we pinned beads in a 5 × 7 cm passage (Supplementary Figure S5a) and as before we compared the cooperative transport of a large load or individually carried small crumbs. The results presented in Supplementary Figure S5b show a similar pattern as above. Note however that here there was no significant difference between the time it took a laden ant to pass through the passage with or without beads (paired t-test: *p*-value > 0.14 Supplementary Figure S5b). This supports the claim that the ants remove the obstacles even though these objects do not significantly restrict the motion of individual ants, including their own. Therefore, clearing small obstructions is useful mainly when ants carry large loads, and provides an advantage in cooperative transport but not in individual transport.

Remarkably, we found that the obstacle clearing behavior of the ants was flexible and was expressed only in a context where this behavior could actually lead to an advantage. To quantitatively assess the degree to which the removal of beads is context sensitive, we quantified the clearing behavior in the presence of either a large food load (Figure 3d) or a pile of crumbs (Figure 3e). In the two conditions, the same type of food was employed, the only difference being the dimension (large or small) of the food items. In these type 2 experiments (see methods) we found that in the latter case, the clearing behavior was significantly reduced. Comparing the number of beads that the ants cleared during the first 30 min of the experiment in each condition, we found that the number of beads cleared in the context of cooperative transport was 32 times greater (*p*-value < 0.00003) than the number of beads cleared when the ants carried the same type of food as individuals (Figure 3f). Furthermore, when the ants were foraging for crumbs, the few beads that the ants eventually moved were dropped after a short displacement of a few millimeters, while the average clearing distance in the context of cooperative transport was more than six times larger (Figure 3g, *p*-value < 10^−8^). Finally, we observed that significantly more ants were recruited to the large load, effectively increasing the ant density there (Figure 3h, *p*-value < 10^−5^). In summary, bead clearing is crucial in the context of cooperative transport but not when ants can retrieve food independently. Consequently, when presented with an identical food type, ants exhibit prominent bead-clearing behavior only if the food is large and requires a joint carrying effort.

### 3.3 Temporal dynamics of bead-clearing experiment

Before going into the microscopic details of the clearing of beads, we first provide an overview of its dynamics at the coarse-grained colony level. We placed a pinned load on an arena full of scattered beads (Supplementary Figure S3). A detailed scoring of the behavior of the first 178 ants recruited to that load was coded (Supplementary Figure S6) and used to highlight the overall dynamics of recruitment and interactions with beads (Figure 4a). After the first ant found the food and realized that it could not retrieve the food alone, it returned to the nest while laying recruitment pheromone markings by frequently lowering its abdomen and touching the floor with the tip of its gaster (Figure 4b). The marking rate is typically around 5.5 Hz (see methods). The recruitment phase eventually led to the accumulation of ants around the load and the initiation of cooperative transport. This also induced a parallel increase in the frequency of inspection of the beads. Bead inspection (“checking behavior”) was defined as ant-bead interaction durations longer than 0.23 seconds, a threshold set by the lowest value between the two peaks of the ant-bead interaction duration distribution (Figure 4c). The bead checking, in turn, was followed by an elevated bead clearing rate (Figure 4d).

### 3.4 Microscopic analysis of clearing behavior

The coarse-grained results presented in the previous section show that an elevated pheromone marking of the ground precedes a sharp increase in recruited ants entering the experimental setup, which in turn is followed by a sharp increase in bead removal. Since the recruitment trail leads the ants directly to the food source, one may suspect that direct contact with a large load that requires cooperative transport is what triggers some of the ants to clear beads. By tracking the bead clearing ants in the overview experiment described in the previous section (Supplementary Figure S6), we found that out of the 18 ants that cleared beads only 8 (45%) actually touched the load. For these 8 ants the time until bead clearing commenced varied greatly (median of 8.05 seconds with a standard deviation of 9.8 seconds). Supplementary Videos S5–S9, Supplementary Figure S11 depict additional instances in which ants cleared beads without coming anywhere near the load. Therefore, it is reasonable to assume that personal knowledge about the presence of the load is not sufficient, and probably not even necessary, for the observed obstacle clearing behavior.

Since a direct experience of the presence of the load is, to the least, not sufficient to induce bead clearing, we tested the importance of socially acquired information by further analyzing type 1 experiments (see methods). We hypothesized that clearing behavior could be triggered by ant sensitivity to increased ant density through the increased interaction rate between ants (Gordon, 1999, 2020), or by interactions of ants with pheromone markings. We tested each of these hypotheses separately at the individual ant level. To test the first hypothesis, we tracked 27 new ants arriving in the experimental setup and counted the number of interactions they experienced with other ants during 10 seconds before interacting with a bead and making a decision to remove the bead (*N* = 14) or not (*N* = 13). Whenever the bead was cleared, it was the first clearing by that ant. The results (Figure 5a) do not show a significant difference between the two conditions (*t-*test: *p*-value > 0.77), suggesting that it is not the ant-ant interaction that elicits clearing behavior.

To test the second hypothesis, we examined 155 cases in which an ant touched a bead. The examination was restricted to a radius of 20 mm (about 7 times the length of the ant) around the selected bead position and a tight time interval of 2 seconds prior to the moment of first contact with the bead. This ensured that our inspections were performed before any decision (either clear or leave) was apparent to the observer. We found that the vast majority (97.2%) of the ants that decided to remove the bead were exposed to a nearby pheromone marking secreted by another ant that happened to pass nearby within the two-second time window. Our results (Figure 5b) show a highly significant correlation (Fisher's exact test: *p*-value < 1*10^−36^) between a nearby marking event and the decision to remove the adjacent bead.

### 3.5 The effect of pheromone marks and recruitment on context sensitivity

Since pheromone markings act as triggers for the clearing behavior, we tested whether the marking rates could explain the context sensitivity described above (Methods and Figures 3d–g). To do this, we examined the recruitment behavior of ants returning from the food. Whereas in the context of a large load, 77.3% of the examined ants (*N* = 17 of 22) deposited a sequence of pheromone marks, only 23.8% of the examined ants (*N* = 10 of 42) engaged in any marking behavior under the control condition (a pile of crumbs from the same food). This increased recruitment led to increased ant densities in the context of cooperative transport [a median of 130 ants in the single large load case vs 7 in the pile of crumbs context (Figure 3h)]. The elevated ant density, in turn, affected the ant-to-ant interaction rates (Figure 5c), leading to significantly more interactions per ant in the single large-load context (*t*-test: *p*-value < 1*10^−12^). Although the rate of interactions between ants is not what triggers clearing behavior (Figure 5a), it does provide an indirect measure of the rate of interactions between a free ant and a nearby pheromone mark recently deposited by another ant. The compounded effect of more ants, each of which lays more pheromone marks, translates into an overwhelming increase in the likelihood that bead clearing will be triggered in the context of cooperative transport.

If the above explanation is correct, as long as the above required conditions for triggering clearing behavior are achieved, we should expect to observe clearing behavior also in an out-of-context scenario: pheromone marks in absence of a large food load. To test this, we offered the ants some canned tuna oil. Although this food is not typical in the natural environment of these ants, it induced an exceptionally high recruitment response, executed by 89.5% of the ants (*N* = 21) that arrived at the food source during the first 10 min. As expected, in this extreme context, some of the ants were triggered to clear the beads even though the group was not involved in cooperative transport at all (see Supplementary Figure S10). This shows that obstacle clearing behavior does not depend directly on cooperative transport. Moreover, this also shows that personal knowledge of cooperative transport is not required before an ant commences clearing beads. The fact that this condition did elicit frequent pheromone markings and that the ants cleared many beads further supports our view that it is indeed social information (pheromone marks) that induces obstacle clearing behavior.

### 3.6 Serial clearers

By keeping track of the ants that cleared a bead in the above mentioned (type 1) experiments, we found that about a quarter of them (*N* = 42 of 167 ants) performed multiple clearings, clearing a total of 289 beads (Figure 5d). To investigate the role of the presence of nearby pheromones in successive clearing events, we analyzed these serial clearers by examining the near-bead area for 2 seconds before each successive bead removal (Figure 5b), as we have previously done for the first clearing event. We found that once an ant has become a clearer, the correlation between proximal pheromone markings and a clearing decision drops significantly (Figure 5e, Fisher's exact test: *p*-value < 2.5*10^−8^. In other words, the ant did not require a pheromone trigger for any bead beyond the first. These findings show that clearing behavior is a specific task to which triggered ants can be allocated (Gordon, 1999). The record holder ant in our data set cleared 64 beads in succession.

Moreover, we found that serial clearers exhibit individual sector preferences. They repeatedly grab beads from a confined area adjacent to the load and then transfer them to approximately the same direction and distance (Figure 6). Such repeated visits may suggest that the ant travels up and down a pheromone gradient which leads it to similar locations. However, an ant can switch its sector preference as can be seen, for example, in Figure 6 in gray, where an ant repeatedly cleared 17 beads from and toward more or less the same direction (mean: –74.4°, SD: 20°) relative to the nest direction (0°), but then shifted to another sector (mean: 138.5°, SD: 26°) around the load and continued clearing 7 more beads. Interestingly, after shifting to the other sector, this ant returned to clear a single bead (bead number 24 in Figure 6) from the previous zone. This anecdotal evidence suggests that personal memory may also be involved in this process. Further study is required in order to settle this issue as well as to determine how specialized this behavior is.

## 4 Discussion

In this study, we present the first report on obstacle-clearing behavior by the longhorn crazy ant *P. longicornis*. When these ants work together to move a large load, only a small portion of the ants actively engage in carrying at any given moment (Ayalon et al., 2021; Gelblum et al., 2015). We discovered that when the terrain is speckled with gravel-sized obstacles, some of the ants that at the moment are not hauling take part in clearing the path. We went on to demonstrate that obstacle clearing is meaningful from a functional perspective, as it significantly reduces the time it takes the ants to transport the food load (Figure 3c). We showed some evidence suggesting that bead clearing was time and energy efficient: the beads cleared were those most likely to interfere with the expected load's motion (Figure 2b) considering the former knowledge of cooperative transport in this species (Fonio et al., 2016), they were carried over relatively short distances (Figure 2c-inset) and dropped at locations that were less likely to coincide with the future location of the load (Figure 2c). We further showed that the microscopic-level mechanisms that trigger an ant to engage in clearing behavior involve short-range exposure to a fresh pheromone mark by another recruiter ant (Figures 4b, 5b). Finally, we found that once activated, the ant has a 25% chance to continue with repeated clearings (Figure 5d), without additional information required (Figure 5e).

Clearing behavior predominantly occurs in regions that lie between the moving load and the nest, these are position where the load can be anticipated to pass shortly thereafter. Such anticipation is possible because, in this species, pheromone deposition is used not only to recruit ants from the nest for load hauling but also to facilitate load navigation (Fonio et al., 2016). Indeed, when crazy ants engage in cooperative transport, they collectively mark the vicinity of the load, blazing the possible retrieval routes between it and the nest entrance. These guiding scent marks grant the ant system its anticipatory capabilities.

Anticipatory bead clearing most likely emerges on the level of the collective and does not require any anticipation by individuals. This is supported by several observations. First, the beads that we used do not interfere with the motion of any individual ant. Second, physically reaching the transported load was not a prerequisite for the removal of beads. In fact, to take on the role of an obstacle clearer, it sufficed that an ant encounter a bead in close proximity to a pheromone mark (Figure 5b). This reliance on pheromone marks, a clearly social signal, is a third observation that supports collective-level emergence. Interestingly, although individual ants do not need direct knowledge of the load's presence to participate in bead clearing, this behavior did emerge specifically in the presence of a large load, where it was most needed. Moreover, bead clearing by the ant group was nest-oriented and fully functional to the transport of the load across the load-to-nest path. We demonstrated how the reliance on pheromone marks can grant the system with this context sensitivity. As mentioned above, in the context of cooperative transport, obstacle clearing is beneficial and sometimes even crucial (Figures 3a–c), for efficient foraging.

It is interesting to compare our findings to previous work on the clearing of debris from ant trails. First, the trail that longhorn crazy ants use during cooperative transport is much more dynamic than classical ant trails (Fonio et al., 2016). Correspondingly, trail clearing from the ephemeral trail employed during cooperative transport occurs on a timescale of minutes [more similar to the paving of sticky paths (Wen et al., 2021)] rather than a timescale of days on stable foraging trails as previously reported (Middleton et al., 2019; Howard, 2001; Bochynek et al., 2017; Rockwood and Hubbell, 1987). Moreover, we show, for the first time, that trail clearing is context-specific as it occurs only when the food at the end of the trail is of the form that benefits most strongly from a cleared path. Most importantly, our work describes both the collective global dynamics and the microscopic single-ant experiences that lead to trail clearing. These provide a first example of the importance of social information, in the form of pheromone markings, as a causal role in the induction of trail clearing (as opposed to clearing by personal experience Bochynek et al., 2019). This allows us to establish trail clearing by crazy ants as an anticipatory behavior that emerges at the level of the colony.

More fundamentally, the emergent colony-level anticipation we describe here relies on representations. Since the superorganism is distributed in its nature, these representations cannot be internal but are rather externalized as pheromone markings in the environment itself (Czaczkes et al., 2015). In the case of longhorn crazy ants pheromone markings serve as external representations of possible future routes for the collectively carried load (Fonio et al., 2016). Generally speaking, representation provides a first step toward decoupling between reality and the cognitive realm. This separation is what differentiates an adaptive system from a truly cognitive one (Pezzulo, 2008). An important property of a cognitive system is its ability to manipulate its representations separately from actions in the real world. Although this aspect was not addressed in this specific work, it is well known that ant trails that are formed by pheromone deposition may interact and affect each other (Beckers et al., 1992).

Finally, it is interesting to fit collective bead clearing into the hierarchical organizational structures (Wilson and Hölldobler, 1988) of the crazy ant colony. Small pebbles do not obstruct the motion of individual ants, and indeed, beads are mainly left uncollected if ants forage on individually carried food crumbs. Nonetheless, individual ants do engage in bead clearing, but they only do so when a specific collective-scale behavior is simultaneously occurring. Indeed, bead clearing only makes sense and mainly occurs when the colony, as a whole, forages for large food items while employing cooperative transport. The ties between bead clearing and cooperative transport are even more intricate as they rely on yet another collective phenomenon—the mass recruitment pheromone trail (Fonio et al., 2016; Czaczkes et al., 2015). It is exactly these pheromone markings that induce bead clearing by individual ants. As noted above, this makes functional sense as pebbles that are adjacent to pheromone markings are most likely to interfere with the carrying team's future motion. Thus, bead clearing provides a striking example of the intertwining of hierarchical structures in the social insect colony. Given the fact that *P. longicornis* ants are nomadic (Trager, 1985; Wetterer et al., 1999; Wetterer, 2008), do not dig nests, and were not known to engage in pebble clearing in their natural environment, it would be intriguing to study how these complex cross-scale inter-dependencies might have evolved.

In sum, our observations show that freshly laid pheromone marks appear to be the triggering stimulus for obstacle-clearing initiation. The presence of a big load alone was not sufficient, nor necessary, for starting obstacle clearing. Conversely, pheromone marks alone did elicit obstacle clearing. We found that obstacle clearing was performed by ants that had never experienced a direct encounter with the big food load, as well as in cases where such load was not even present, just in response to pheromone marks alone. These results strongly suggest that the individual ants do not possess an internal representation of the final goal of obstacle clearing. On the other hand, the goal-directedness of obstacle clearing appears to emerge at the ant group level from collective cognition.

## Data Availability

The raw data supporting the conclusions of this article will be made available by the authors, without undue reservation.
