# Supplementary material for: Ants engaged in cooperative food transport show anticipatory and nest-oriented clearing of the obstacles surrounding the food: goal-directed behavior emerging from collective cognition
Source: Front Behav Neurosci. 2025 Jun 13;19:1533372. doi: 10.3389/fnbeh.2025.1533372 (PMC12202405; doi:10.3389/fnbeh.2025.1533372)
Supplement: Supplementary file 10 [file Data_Sheet_1.pdf]

Ants engaged in cooperative food transport show anticipatory and nest-oriented clearing of the obstacles surrounding the food: goal-directed behavior emerging from collective cognition

Ehud Fonio[1*], Danielle Mersch[1], and Ofer Feinerman[1]

[1]*Department of Physics of Complex Systems, Weizmann Institute of Science, Rehovot, Israel*
[*]*Corresponding author : ehud.fonio@weizmann.ac.il*

Supporting information

Video S1: This is part of our first documentation of collective obstacle clearing during cooperative transport. In the video, the ants carry a dead mantis toward their nest (to the right), during which other ants occasionally remove gravel (marked by red circles) from the vicinity of the transported load.

Video S2: In contrast to food transport, when ants join together to encircle the food load and carry it to the nest, obstacle clearing was typically carried out by single ants. This video shows an extreme example of an ant singly clearing an obstacle (piece of glass) much larger than its body size.

Video S3: Marking behavior during recruitment. In this video, the first ant that found the large food load lays a series of markings while returning to the nest. Here, the pheromone is represented by purple circles, which gradually grow and fade from the position of each marking event, simulating the evaporation of the pheromone (see more details in the methods).

Videos S4-9: Obstacle clearing without approaching the food load first. Each of these videos shows the area around an ant arriving to the experimental set-up and clearing a bead without having ever got before to the food load. These examples also support our claim that the ants involved in removing obstacles do not need to have personal information about the existence of a large load that requires cooperative transport.

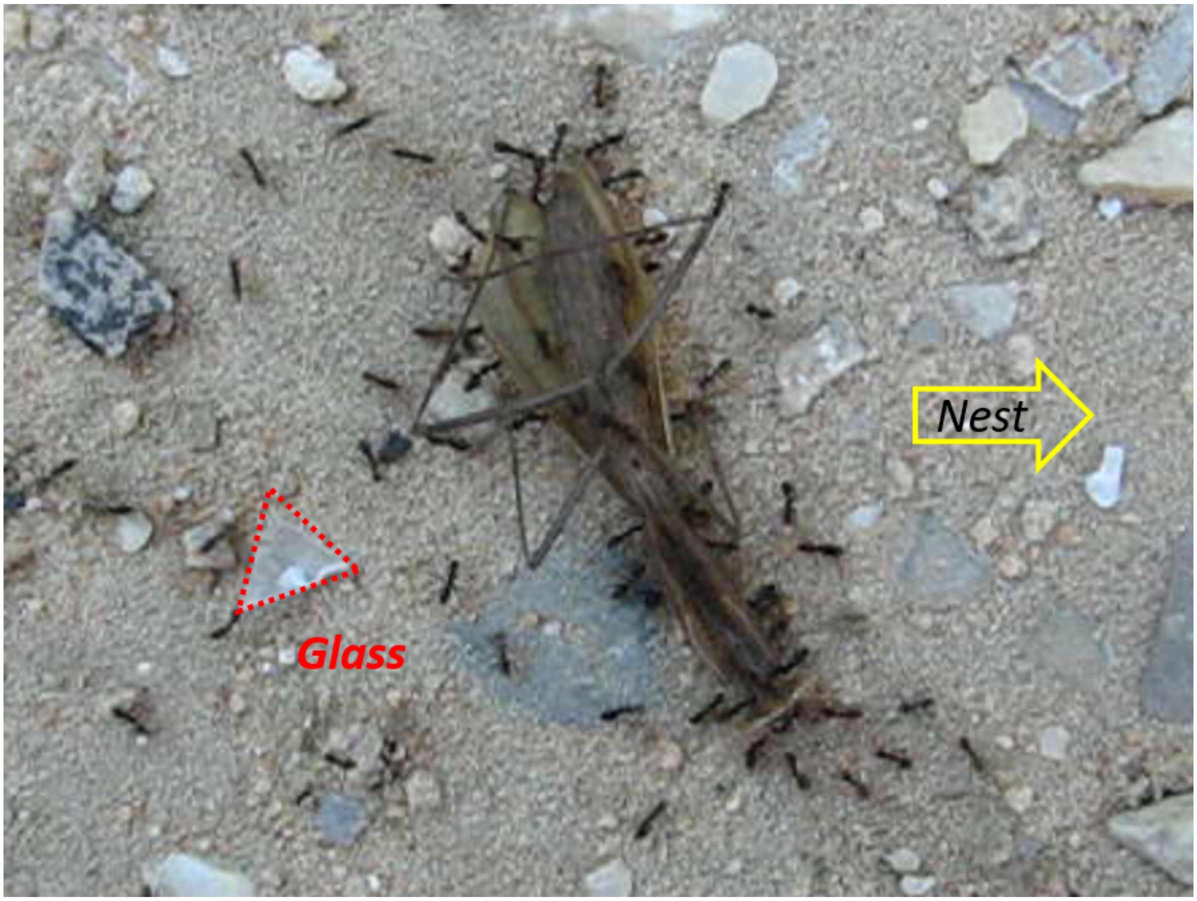

Figure S1: A single ant clears a large piece of glass (see also Video S2).

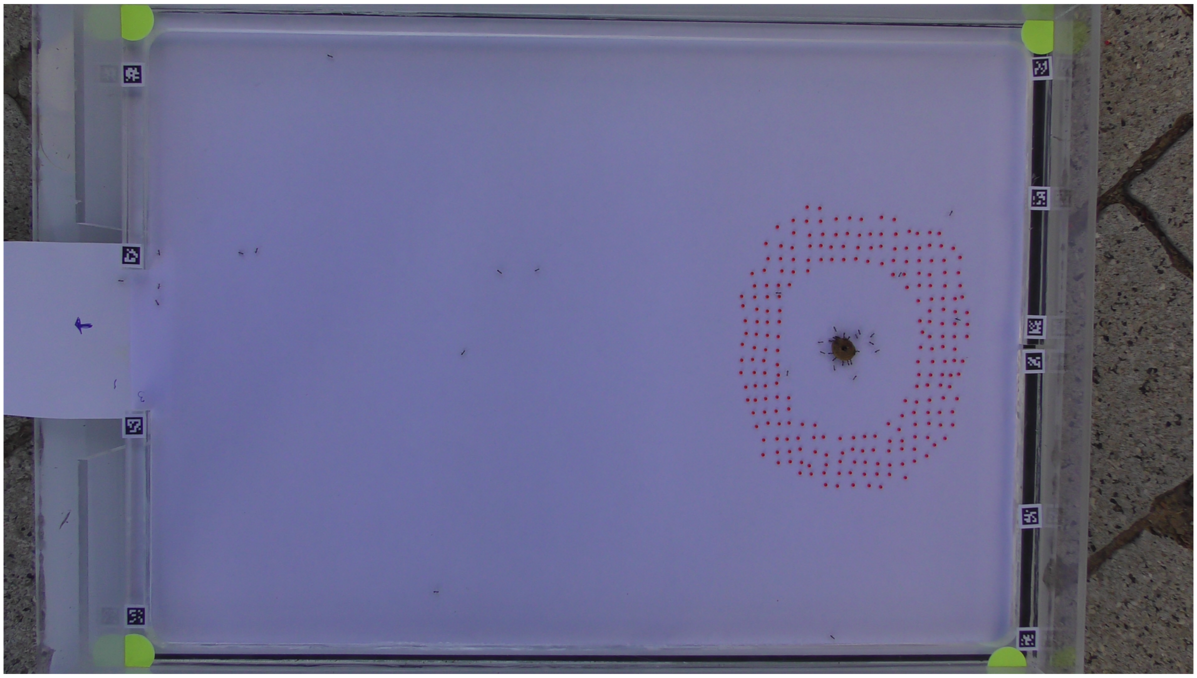

Figure S2: The experimental set-up: bar-codes on the edges allowed specifying the bead positions and measuring clearing distances.

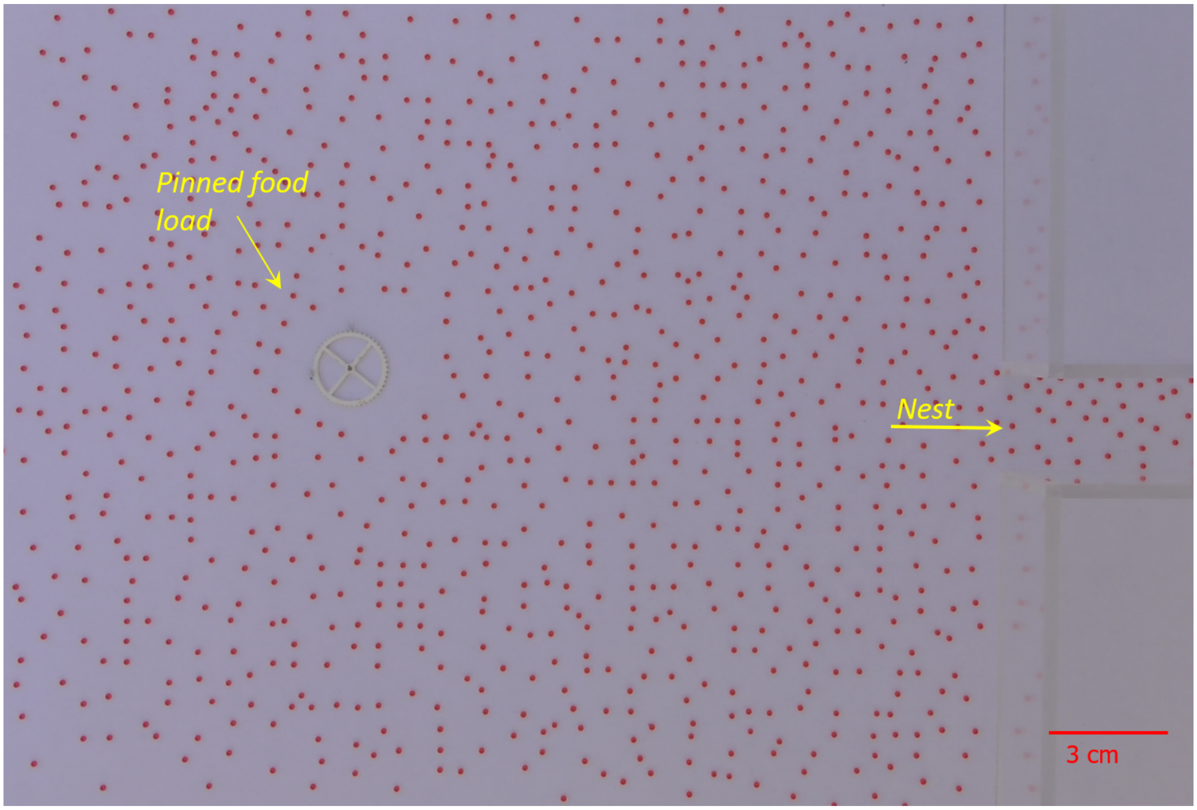

Figure S3: The experimental set-up: A silicon ring coated with food scent, on a pin (rotation allowed) in an arena full of beads.

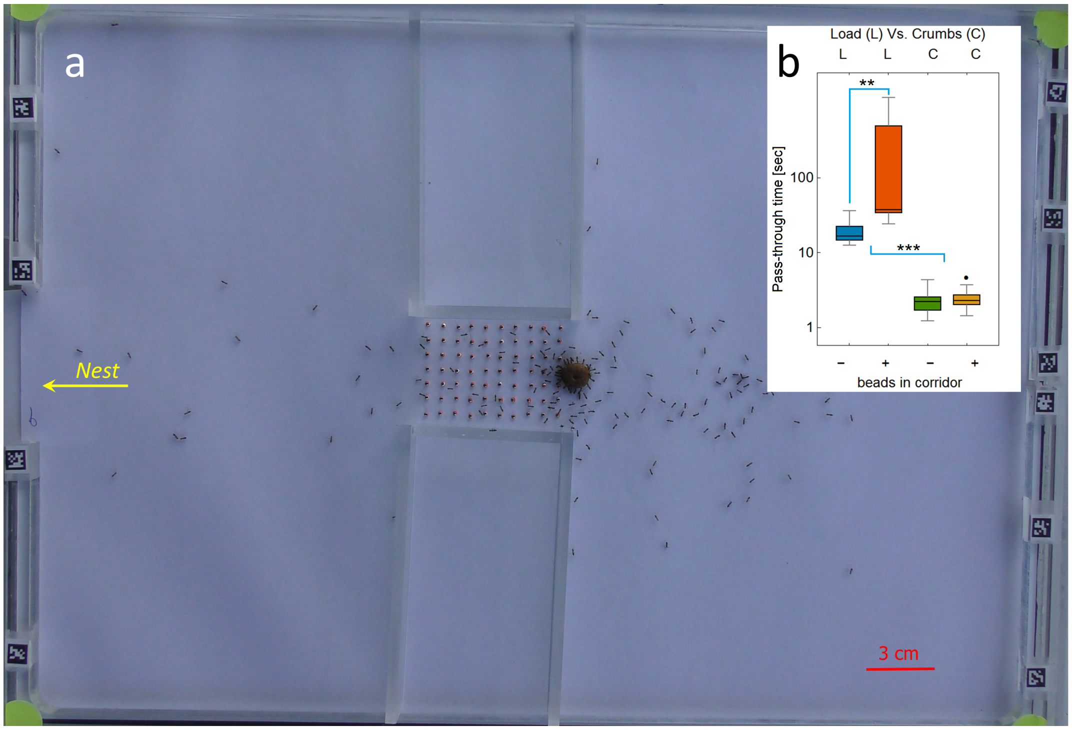

Figure S4: Clearing function (type 2 experiments). a) An experimental set-up with a 5x7 cm passage in which an array of beads were pinned to the floor. A load (brown object) was positioned on the far side relative to the nest such that the ants had to transport the food load through the passage. b) Box plots of the pass-through time of the transported load across a passage with beads (N=7, median=53.5, SD=23.2, in orange) or without beads (N=8, median=16.8, SD=7.9, in blue). In addition the same tests have been done with single ants carrying small crumbs (as used in the context tests described in Figure 3f-g) to measure the effect of beads on individual ants (N=20 for each condition). The pass-through time for the empty passage is median=2.2, SD=0.76 (in green) and median=2.3, SD=0.79 for the passage with beads (in yellow). Asterisks highlight significant differences (** $p < 0.01$, *** $p < 0.001$).

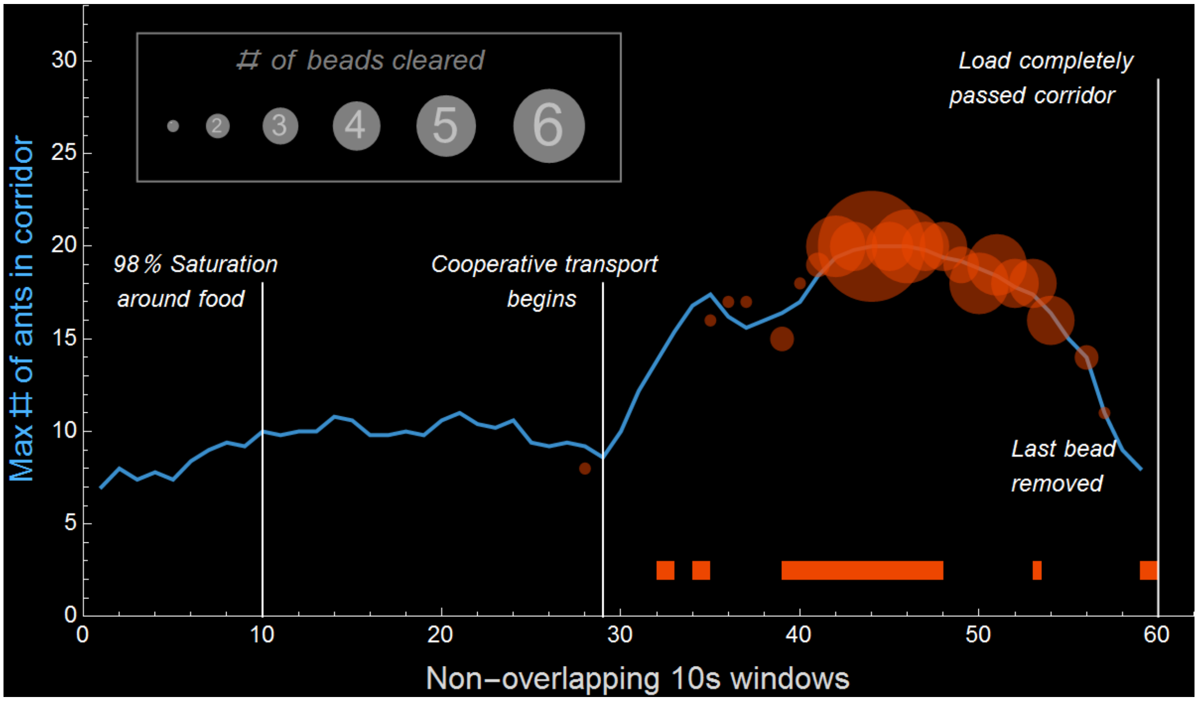

Figure S5: An example of the clearing dynamics in one of the corridor experiment, where the ants had to first clear a corridor full of beads (N=73) before they could make the load pass through it. In each non-overlapping time window of 10 seconds the maximal number of ants in the corridor was recorded (blue line). In addition, the number of beads that were cleared within each time window was also coded and is presented as an orange disk, whose size is proportional to that number (see scale in gray). Time intervals in which at least part of the load was inside the corridor are depicted as orange bars at the bottom of the plot, showing repeated attempts to cross the corridor. Only after the last bead was removed the ants managed to finally get the load to the other side, and they did within a short time range in agreement with the 'pass-through time' results presented in Figure 3c.

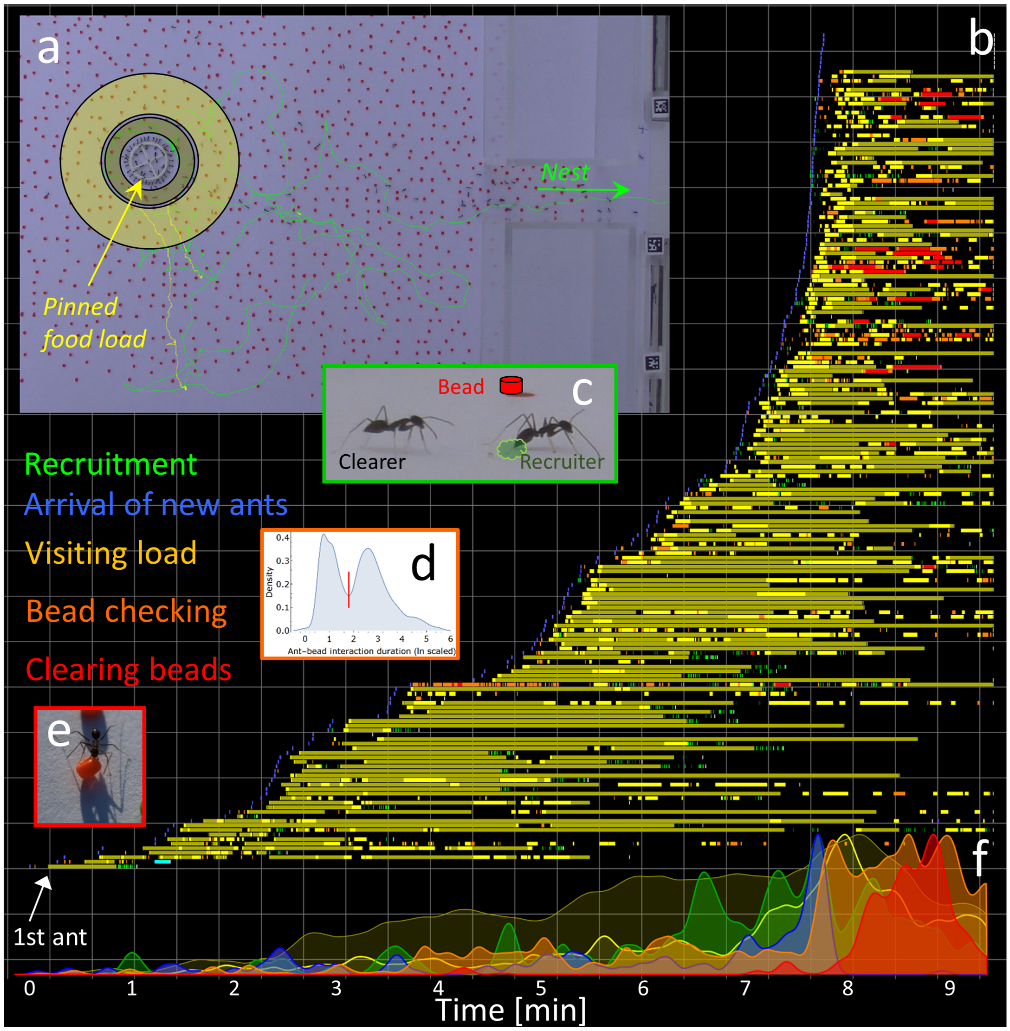

Figure S6: Detailed ethogram of recruitment and clearing behavior: (a) The experimental arena full of beads, a pinned load (white), the proximate area around the load (in yellow), and 2 trajectories of ants (green lines) that were eventually involved with bead clearing (yellow lines). (b) The dynamics of mass recruitment and clearing behavior: Each horizontal bar represents the behavioral description of an ant. The ants are sorted according to arrival time to the experimental set-up (from bottom to top, in blue). Other scored behaviors are: staying near the load (yellow ring in a), marking behavior (green), checking beads (orange) and clearing beads (red). (c) A close-up picture showing a recruiter ant depositing a pheromone mark by touching the ground with the tip of its gaster. (d) A density distribution of ant-bead interaction duration (log transformed) showing a clear bi-modality. Interactions that belong to the right component were defined as checking behavior. (e) A picture of an ant clearing a bead. (f) A normalized density plot of the various behaviors mentioned above (same color code) summarizing the intensity of the different coded behaviors along time.

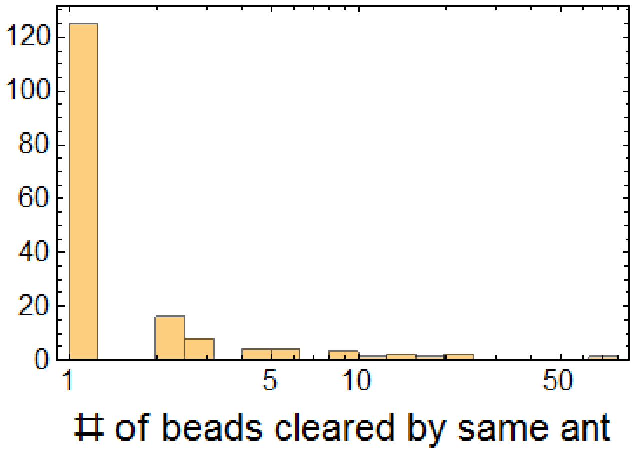

Figure S7: Number of beads cleared by the same ant.

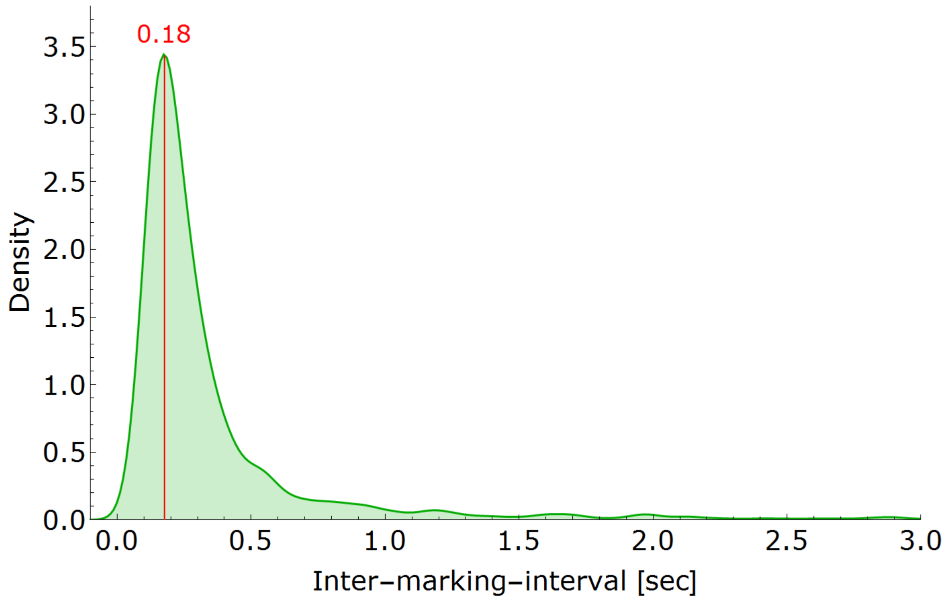

Figure S8: Inter marking interval: The distribution of time duration between successive marking events (N=2,351). This distribution was created by using the the detailed data collected from one of the experiments (Figure S5)

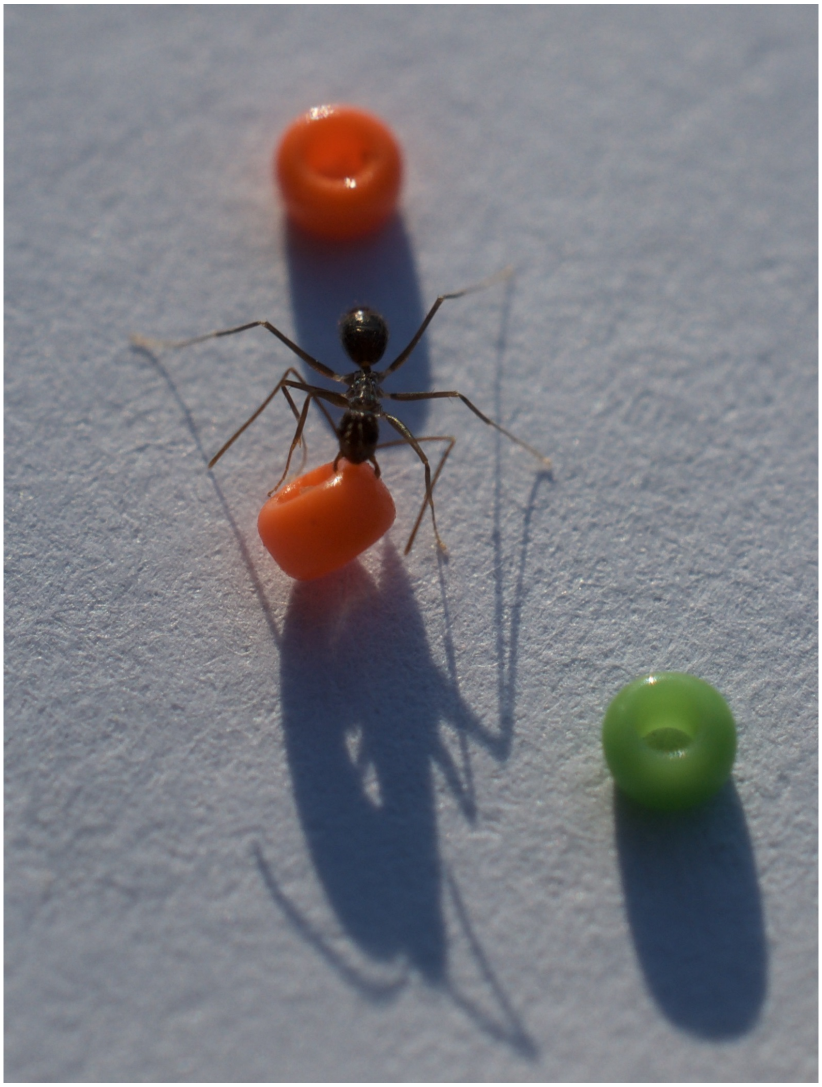

Figure S9: The original picture from which figure 1b was created.

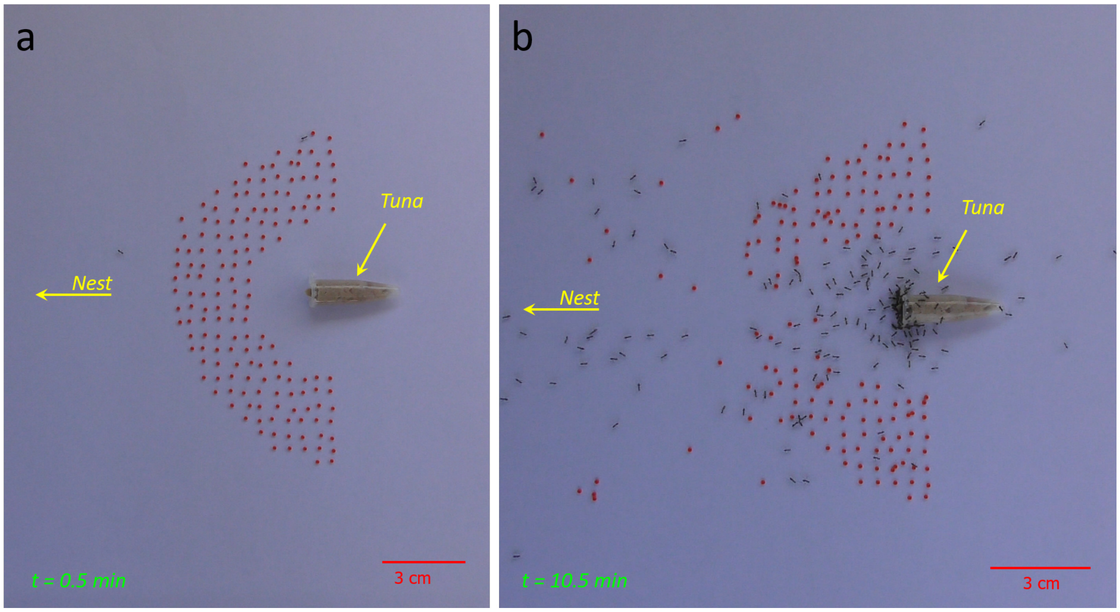

Figure S10: Clearing beads out of the context of cooperative transport by using highly attractive food (tuna oil). The strong recruitment involved many scent marks and recruited ants followed by extensive bead clearing. a) A frame from the beginning of the experiment. b) A frame taken 10.5 min into the same experiment.

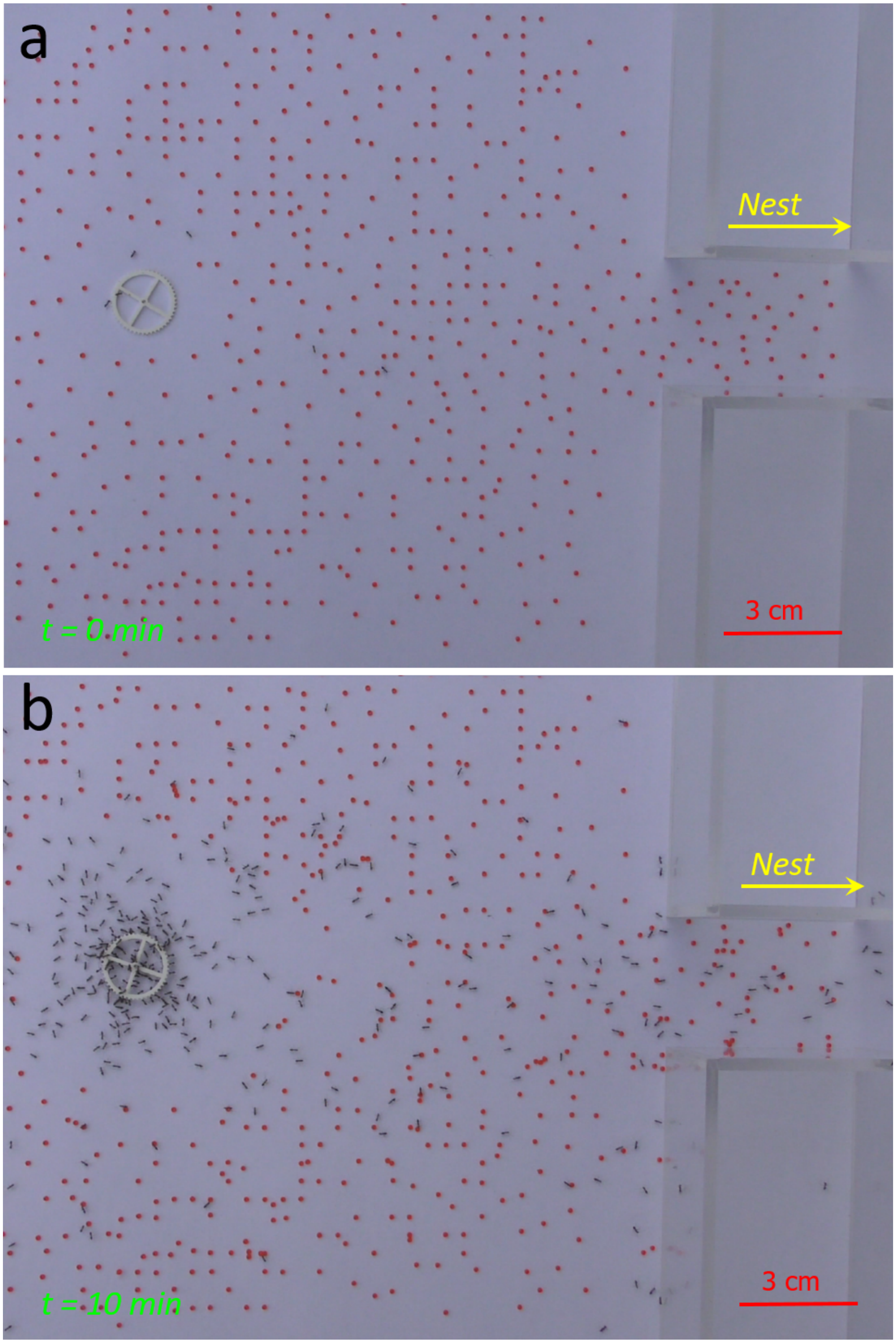

Figure S11: Ants arriving from the nest and clearing beads from the entrance to the arena (the corridor on the right side of each frame) without approaching the food load first. a) A frame from the beginning of the experiment. b) A frame taken 10 minutes into the experiment, after many beads were cleared, including from the entrance to the experimental set-up.
